# A deep-learning approach for segmentation of liver tumors in magnetic resonance imaging using UNet++

**DOI:** 10.1186/s12885-023-11432-x

**Published:** 2023-11-03

**Authors:** Jing Wang, Yanyang Peng, Shi Jing, Lujun Han, Tian Li, Junpeng Luo

**Affiliations:** 1 Department of General medicine, The First Medical Center Department of Chinese PLA General Hospital, Peking, 100039 China; 2 Department of Radiology, First Medical Center of General Hospital of People’s Liberation Army, Peking, China; 3 Department of Oncology, Huaihe Hospital, Henan University, Kaifeng, 475000 China; 4 Department of Radiology, State Key Laboratory of Oncology in South China, Collaborative Innovation Cancer for Cancer Medicine, Sun Yat-sen University Cancer Center, Guangzhou, 510030 China; 5 School of Basic Medicine, Fourth Military Medical University, Xi’an, 710032 China; 6 Translational Medical Center of Huaihe Hospital, Henan University, 115 West Gate Street, Kaifeng, 475000 China; 7 Academy for Advanced Interdisciplinary Studies, Henan University, Zhengzhou, 450046 China

**Keywords:** Hepatocellular Carcinoma, Magnetic resonance imaging, UNet++, Segmentation, Deep learning, Radiomics

## Abstract

**Objective:**

Radiomic and deep learning studies based on magnetic resonance imaging (MRI) of liver tumor are gradually increasing. Manual segmentation of normal hepatic tissue and tumor exhibits limitations.

**Methods:**

105 patients diagnosed with hepatocellular carcinoma were retrospectively studied between Jan 2015 and Dec 2020. The patients were divided into three sets: training (n = 83), validation (n = 11), and internal testing (n = 11). Additionally, 9 cases were included from the Cancer Imaging Archive as the external test set. Using the arterial phase and T2WI sequences, expert radiologists manually delineated all images. Using deep learning, liver tumors and liver segments were automatically segmented. A preliminary liver segmentation was performed using the UNet + + network, and the segmented liver mask was re-input as the input end into the UNet + + network to segment liver tumors. The false positivity rate was reduced using a threshold value in the liver tumor segmentation. To evaluate the segmentation results, we calculated the Dice similarity coefficient (DSC), average false positivity rate (AFPR), and delineation time.

**Results:**

The average DSC of the liver in the validation and internal testing sets was 0.91 and 0.92, respectively. In the validation set, manual and automatic delineation took 182.9 and 2.2 s, respectively. On an average, manual and automatic delineation took 169.8 and 1.7 s, respectively. The average DSC of liver tumors was 0.612 and 0.687 in the validation and internal testing sets, respectively. The average time for manual and automatic delineation and AFPR in the internal testing set were 47.4 s, 2.9 s, and 1.4, respectively, and those in the external test set were 29.5 s, 4.2 s, and 1.6, respectively.

**Conclusion:**

UNet + + can automatically segment normal hepatic tissue and liver tumors based on MR images. It provides a methodological basis for the automated segmentation of liver tumors, improves the delineation efficiency, and meets the requirement of extraction set analysis of further radiomics and deep learning.

**Supplementary Information:**

The online version contains supplementary material available at 10.1186/s12885-023-11432-x.

## Introduction

Since radiomics-related research is becoming increasingly important for hepatocellular carcinoma research, accurate and efficient tumor segmentation has become increasingly critical. As tumors are heterogeneous, MRI imaging sequences vary, imaging protocols vary, and radiologists with varying degrees of experience define liver tumors manually [1], which is not easy to repeat [2]. It is imperative that clinicians utilize artificial intelligence technology to solve this problem [3]. Standardized image analysis and reporting can reduce the inconsistency in image interpretation. At present, Liver Reporting and Data System is one of the most widely used systems [4, 5]. Nevertheless, the workload per case read by a diagnostic radiologist is increased with the use of the system. Semiautomatic image segmentation [6] has led to an improvement. However, its accuracy is low, and its effectiveness depends directly on the doctor’s experience. Automated segmentation [7] has become a research hotspot in recent years. Nevertheless, most of the studies in these two categories lack external validation.

Medical image segmentation has undergone three stages of development, involving traditional image segmentation, machine learning, and deep-learning techniques [8], Radiomics based on this has been widely used in clinical prediction of tumor staging [9]. Deep learning techniques have revolutionized medical imaging across various domains. In lung nodule detection [10], CNNs analyze CT scans for early cancer detection. Prostate segmentation benefits from U-Net’s precision in MRI analysis [11], Brain tumor segmentation employs CNNs for accurate tumor delineation in MRI scans [12]. The type of deep-learning model determines the content and accuracy of the segmentation results. In the field of liver and liver tumor segmentation, several recent studies [13–17] report computed tomography (CT)–based liver segmentation using deep learning methods, and the segmentation performance appears to be good [18]. Hepatocellular carcinoma is currently diagnosed and evaluated using magnetic resonance imaging (MRI), which is more sensitive and specific than CT for lesions with diameter < 3 cm and equivalent to CT for larger lesions [19]. Applying deep learning to MRI-based liver segmentation faces challenges due to MRI’s diverse contrasts, artifacts, and tissue variations. Unlike CT, MRI lacks consistent intensity values, requiring models to adapt to variations. Additionally, the soft tissue contrast in MRI presents unique segmentation complexities. Addressing these variations and contrasts is pivotal for accurate MRI-based liver segmentation. Few reports are published on the semantic segmentation of livers and liver tumors using MRI deep-learning model frameworks [20]. The application of UNet + + has exhibited great potential in medical imaging, such as fully automated tumor segmentation and response assessment in brain imaging [21, 22] and prostate imaging [23]. The strength of this algorithm lies in the divide and conquer solution rather than feature fusion. However, its performance on segmenting hepatocellular carcinoma is unclear. In this study, we aimed to develop a deep learning model using UNet + + that can automatically segment the liver and liver tumor from multisequence MR images.

## Materials and methods

### Clinical data

This study examined the Picture Archive and Communication System (PACS) at Sun Yat-Sen University Cancer Hospital to identify individuals with hepatocellular carcinoma (pathologically or clinically diagnosed [24]), who underwent MRI examination before treatment between Jan 2015 and Dec 2020. Patients treated through radiofrequency ablation or microwave ablation [25] were selected. The Ethics Committee of Sun Yat-Sen University approved the study (SLB2022-047-02). Informed consent has been obtained from the patients/participants in this study.

### Enrollment criteria

The inclusion criteria were as follows: (1) patients with clinical or pathological diagnosis of hepatocellular carcinoma, (2) number of tumors ≤ 3, (3) age of patients: at least 18 years, (4) implementation of multiphase dynamic contrast-enhanced MRIs, (5) Diagnosis: Patients included in our study were diagnosed with hepatocellular carcinoma (HCC) based on clinical and radiological findings. The diagnosis was confirmed using standard clinical criteria and imaging modalities, (6) Imaging Data: Patients had undergone magnetic resonance imaging (MRI) examinations for liver evaluation, specifically with multisequence MR images that included arterial phase and T2-weighted images, (7) Availability of Annotations: The inclusion criteria required that expert radiologists had manually annotated the MR images to provide ground truth segmentations of liver tumors. These annotations served as the reference standard for evaluating the performance of the UNet + + model. The exclusion criteria were as follows: (1) metastasis of hepatocellular carcinoma, (2) no clear boundary between the liver tumor and surrounding tissue, (3) poor image quality or motion defects, (4) use of Gd-EOB-DTPA as the contrast agent in MRI, (5) Incomplete or Poor-Quality Imaging Data, (6) Missing Annotations,7) Maging artifacts: Patients with MR images affected by significant imaging artifacts that could interfere with accurate tumor segmentation were excluded.

In total, 105 patients were enrolled in the study, including 96 males and 9 females. The baseline conditions of the patients are shown in Table 1. Clinical characteristics such as age, gender, and the number of tumors revealed in MRI were analyzed before ablation treatment. The number of tumors and their maximum diameter were obtained from electronic medical records.

**Table 1:** Patient Baseline Information

Parameter	Numerical value
Patients Numbers	105
Age(mean ± standard deviation)	58 ± 11
Gender( Male/Female)	96/9
Tumor Numbers	198
single/multiple	51/54
Median number of tumors	2
Imaging-based diagnosis of hepatocellular carcinoma	95
Pathologically diagnosed hepatocellular carcinoma	10
Tumor diameter (cm)	2.2 ± 1.5
Tumor location (right lobe/left lobe/both sides)	60/20/25

Prior to ablation, the last enhanced MR images were selected, and all images were acquired when the patient was fully inhaled in a supine position. MRI was performed using Discovery MR 750 (GE Healthcare, Milwaukee, WI, USA). The T1WI was performed using gradient echo with the repetition time (TR) of 3.5–4.0 ms, echo time (TE) of 1.5–2.0 ms, field of view (FOV) of 300 × 400 mm, matrix of 256 × 256, flip angle of 10°, thickness of 5 mm, and crossing gap of 1–2 mm. The T2WI was performed using spin echo with TR of 2500 ms, TE of 90 ms, FOV of 300 × 400 mm, matrix of 384 × 256, flip angle of 20°, thickness of 5 mm, and crossing gap of 1–2 mm. The diffusion-weighted imaging (DWI) obtained 2 b values (0 and 800 s/mm^2^) with TR of 2600 ms, TE of 59.5 ms, FOV of 300 × 400 mm, matrix of 128 × 128, flip angle of 90°, thickness of 5 mm, and crossing gap of 1–2 mm. The contrast agent was gadoterate meglumine (0.1 mmol/kg). A series of images was acquired after intravenous injection of the contrast agent at three different time points: the arterial (25–30 s), portal venous (60–70 s), and delayed (3 min) phases. The acquired MR images were uploaded to PACS.

### Manual segmentation of the MR images

Two radiologists with 5 or 7 years of experience in reading upper abdominal magnetic resonance (MR) images independently evaluated the imaging features irrespective of clinical data. Using 3D-slier software [26] (https://www.slicer.org/, version: 4.10.2 r28257, Windows 11 professional edition), they manually segmented 3D images of whole liver and tumors at the arterial phase and T2WI MR images. On the subject MR images, a semi-automated segmentation algorithm was applied using the flood fill algorithm of the 3D Slicer software. It was necessary to install the Segment Editor Extra Effects extension through the extension’s manager prior to installation. Once the DICOM module has loaded data images, observers are able to identify the location of the HCC. Mouse cursors were used to add nodes around the tumour region. Afterward, flood fill effects were activated, and ROI were segmented according to similar voxel intensities. During the finalization phase, the segmented tumour was manually edited as a semi-automated segmentation process. Consensus was achieved through discussions and consensus meetings in case of discrepancies. The masked image was determined by overlaying individual delineations, creating a consensus delineation considered the gold standard for model training and evaluation.

### Image preprocessing

MRI images underwent intensity normalization, resizing to a common resolution, and window-level adjustment. Voxel size was standardized through interpolation. Image registration for T1 arterial phase and T2WI images was performed using rigid registration to align them for accurate segmentation. For this study, the voxel size of the modified MR images was uniformly set to 1.0 mm × 1.0 mm × 5.0 mm. To register the images of T1 arterial phase and T2WI from the same case, the SimpleIT [27] (http://www.simpleitk.org) registration tool was used. The pure black background frame with voxel value of 0 in the original MR image was removed to reduce invalid training caused by the black background (voxel value = 0). Only the portion of the image containing human tissue was retained to reduce invalid training. Image voxel values were sorted from small to large, and the entire MRI voxel values varied from 0 to 99.8%. The details of the hardware and software configurations used in this study are given in the Supplementary Tables 1 and 2.

### Development of deep neural network model

**Fig. 1:**
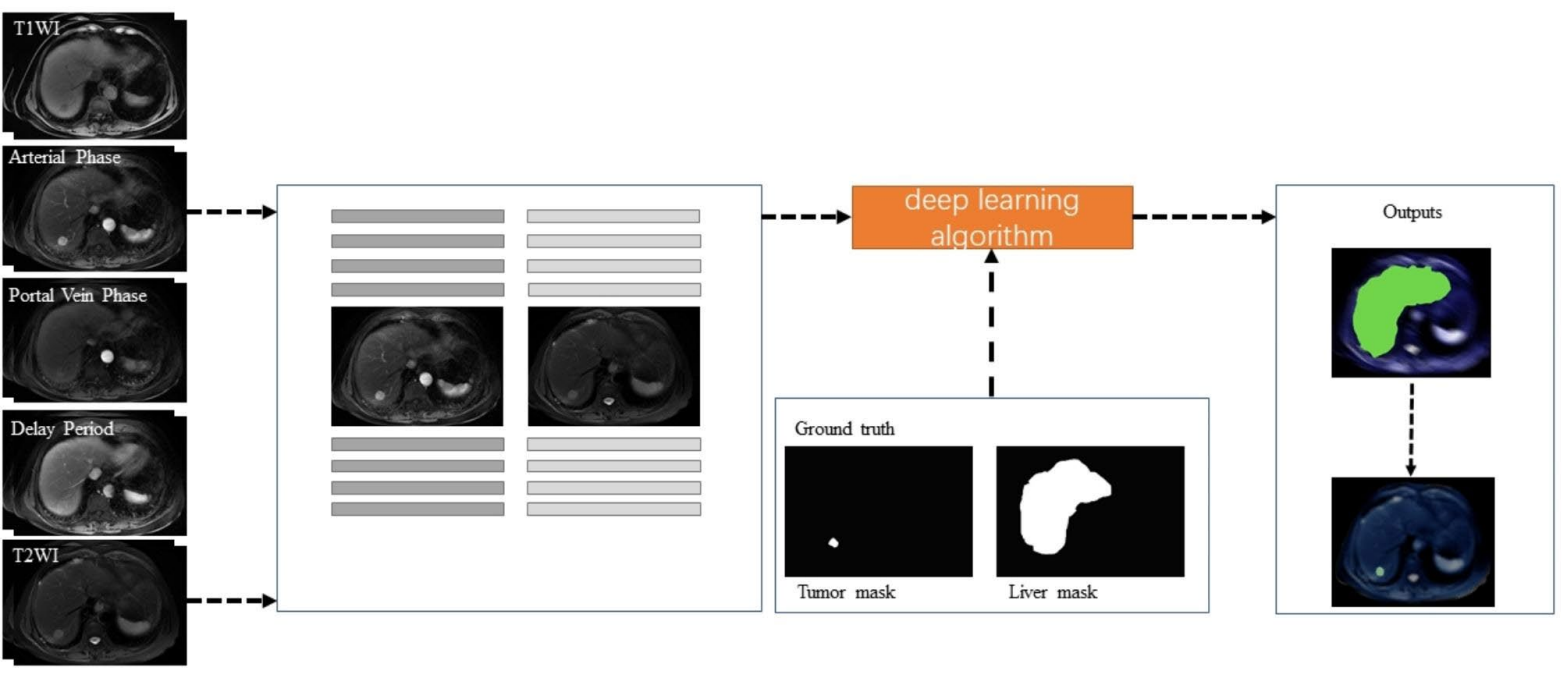
Automated segmentation process. To make masks, the MR arterial phase and T2 images of each patient were extracted from the training set and were manually delineated layer by layer to identify the liver and tumor. Ground truth is provided by these masks. A model for automatic delineation is trained based on the ground truth. Next, the validation and test sets were assembled and input in the trained model, and finally layer by layer, the images with masks were obtained

The two-stage semantic segmentation utilized UNet + + for liver and tumor delineation. In the first stage, the liver was segmented using 2D slices. Then, the liver mask was input for tumor segmentation using both 2D and 3D UNet++. Axial slices were processed independently during evaluation to provide accurate 2D predictions (Fig. 1). From these models, slices using the gold standard in segmentation were learned, and two-dimensional (2D) segmentation of the liver and tumor was generated (Supplementary Fig. 1). The algorithm was applied to each axial slice during the evaluation, and the resulting 2D segmentation was superimposed to produce a 3D segmentation of the entire image sequence.

### Model training

Random scaling, rotation, and flipping altered image orientation. Affine elastic transformation simulated tissue deformations. Noise injection mimicked real-world imperfections. Cropping focused on relevant regions, enriching training data. These techniques enhanced model robustness by introducing diverse variations and reducing the risk of overfitting. The specific data augmentation methods include random scaling (the range of the scaling ratio is 0.8–1.2), random rotation (the range of the rotation angle is ± 180°), random flip, random affine elastic transformation, random noise, random cropping, etc. For the first-stage model, the size of the cropped image block during training was 224 × 224. For the second-stage model, the size of the cropped image block during training was 128 × 128. During training, the loss functions used were the cross-entropy loss function and DSC loss function. The solver used was Adam. The initial learning rate was set as 0.001, and the L2 regular term coefficient was set as 0.0001.

### Results handling after segmentation

After the initial segmentation, the predicted liver mask results were subjected to a post-processing stage. This involved applying morphological operations, specifically dilation and erosion, to enhance the accuracy of the masks. The dilation operation expanded the segmented areas slightly, while the erosion operation shrunk them back, resulting in smoother and more coherent liver masks. This post-processing step helps mitigate any potential inconsistencies or noise introduced during the initial segmentation process. The predicted liver mask results of each MR image were analyzed in accordance with the first-stage network. Domains with the largest volume were retained, and those with smaller volumes were deleted. Small voids were eliminated via morphological closure operations on the remaining connected domains. Finally, a final liver mask prediction was obtained. For the second-stage network, the predicted liver mask results of each MR image were analyzed by the connected domain, and the independent connected domains were distinguished. The mean probability of tumor class in the last activation layer from each independent connection domain is shown in Supplementary Figs. 2 and 3.

### Statistical analysis

The performance of automated segmentation was evaluated by comparing fully automated segmentation with manual segmentation of liver tumors using the gold standard. The quality of the segmentation was measured using the metrics defined in the liver tumor segmentation challenge, including the Dice similarity coefficient (DSC) [28]. To evaluate the performance of the algorithm in the detection task, the sensitivity (percentage of correctly detected lesions) and average false positivity rate (AFPR) [29] were calculated. Consequently, if the automated segmentation does not overlap with the manual segmentation that uses the gold standard, the result of automated segmentation was regarded as false positive. Additionally, the DSC of each tumor was measured to assess segmentation quality. The concordance correlation coefficient [30] was calculated using MedCalc (version 20.027, 64-bit, www.medcalc.org) to compare the consistency of the true and predicted volumes of liver tumors, as well as the time required for fully automated and manual segmentations. The volumes of liver tumors obtained in manual segmentation of external test set and automated segmentation were compared using Prism (Version 9.0.0, 64-bit, www.graphpad.com).

## Results

### Characteristics of patients

A total of 105 patients with multisequence MR images containing 198 lesions were included in this study. According to a ratio of 8:1:1, the entire data set was randomly divided into 83, 11, and 11 training, validation, and internal testing groups, respectively. Table 1 summarizes the baseline characteristics of the patients in the training and internal testing groups. The average age of the patients was 58 years. The majority of patients had multiple lesions. Most of the diagnosis was based on clinical evidence. The average diameter of the tumors was 2.2 cm, and most of them were located in the right lobe of the liver.

### Liver segmentation

The semantic segmentation of the liver exhibited superior performance in the validation and internal testing groups, and the automatic delineation time was significantly shorter than the manual delineation time (Supplementary Table 3). In the validation set, the average DSC value was 0.91, and the average time saved by automatic delineation was 180.7 s. In the internal testing set, the average DSC value was 0.922, and the average time saved by automatic delineation was 168.1 s. In both the validation and internal testing sets, automated segmentation of the liver exhibited a good performance, resulting in an increase in segmentation efficiency and reduction in segmentation time. Supplementary Figs. 4 and 5, respectively, show the worst and best cases of automated segmentation of the liver in this study.

### Tumor segmentation

The DSC was calculated based on each patient (Supplementary Table 4). Although the automatic delineation saved 44.5 s, the average DSC was only 0.526, and the false positivity rate was 1. This indicated that on an average, one extra false-positive lesion was segmented per patient. Patient no. 2 had two lesions, whereas only one lesion was automatically segmented. The occurrence of false-positive lesions can be attributed to several factors, including variations in image quality, tissue appearances, and complex tumor shapes. The model might misinterpret normal structures or artifacts as tumors, leading to these false positives. Further analysis is needed to refine the model’s sensitivity and minimize these instances.

Case-based statistics would result in lower DSC values. Because some patients had multiple tumors, the segmented tumors were statistically analyzed, and the results of each tumor were separately evaluated. In Supplementary Tables 5, a DSC of 0 indicated a missed detection; it is most often associated with tumors of diameter < 2 cm or unclear segmentation of the tumor from the surrounding normal liver tissue. In the internal testing set, the average DSC value was 0.61. The size of multiple tumors in the internal testing set was relatively small, which may contribute to the decrease in the average DSC value. Supplementary Figs. 6 and 7, respectively, show the worst and best cases of automated segmentation of the tumors in this study.

Furthermore, the algorithm for segmenting liver tumors was externally validated in this study using the public liver cancer data set of TCIA [31]. The data set consisted of 97 patients, among which, more than 40 underwent MRI examination. The patients having MR images of poor quality were excluded from the analysis. The algorithm was validated using a public liver cancer dataset from TCIA, comprising 9 cases. MR image quality varied, but the algorithm demonstrated consistent tumor segmentation performance across different data sources. This external validation confirmed the model’s applicability beyond our dataset. (Supplementary Table 6). The average time saved by automatic delineation was 25.3 s; the average DSC value per case was 0.611, and the AFPR was 1.6. However, in case no. 4, only one of the two lesions was segmented. In case nos. 6 and 5, three and one extra lesions were segmented, respectively. Each of the remaining cases had two extra lesions segmented. If the segmentation was analyzed based on each tumor, the results are shown in Supplementary Table 7. In total, 10 lesions were automatically segmented from 9 patients, with an average DSC of 0.687. Supplementary Fig. 8 shows the results of automated segmentation of the liver tumors in patient no. 1 in the external test set.

**Fig. 2:**
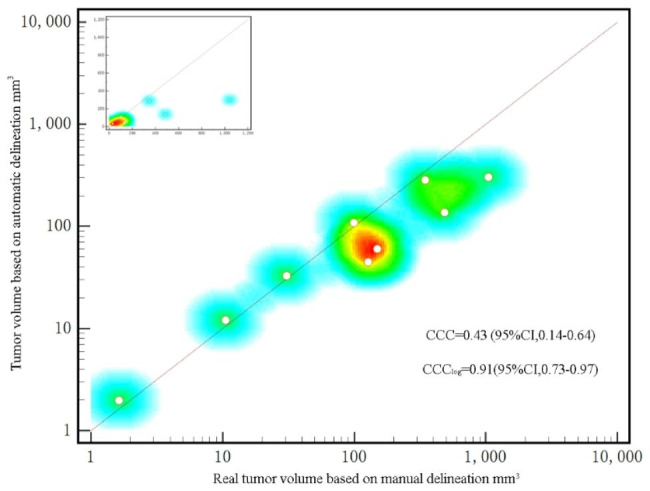
Scatter plot of actual volume vs. predicted volume of liver tumors in the external test set. The concordance correlation coefficient after the logarithm of the actual and predicted volumes was 0.91, indicating that the volume of the 9 livers in the external test set obtained via automated segmentation was close to the actual volume

**Fig. 3:**
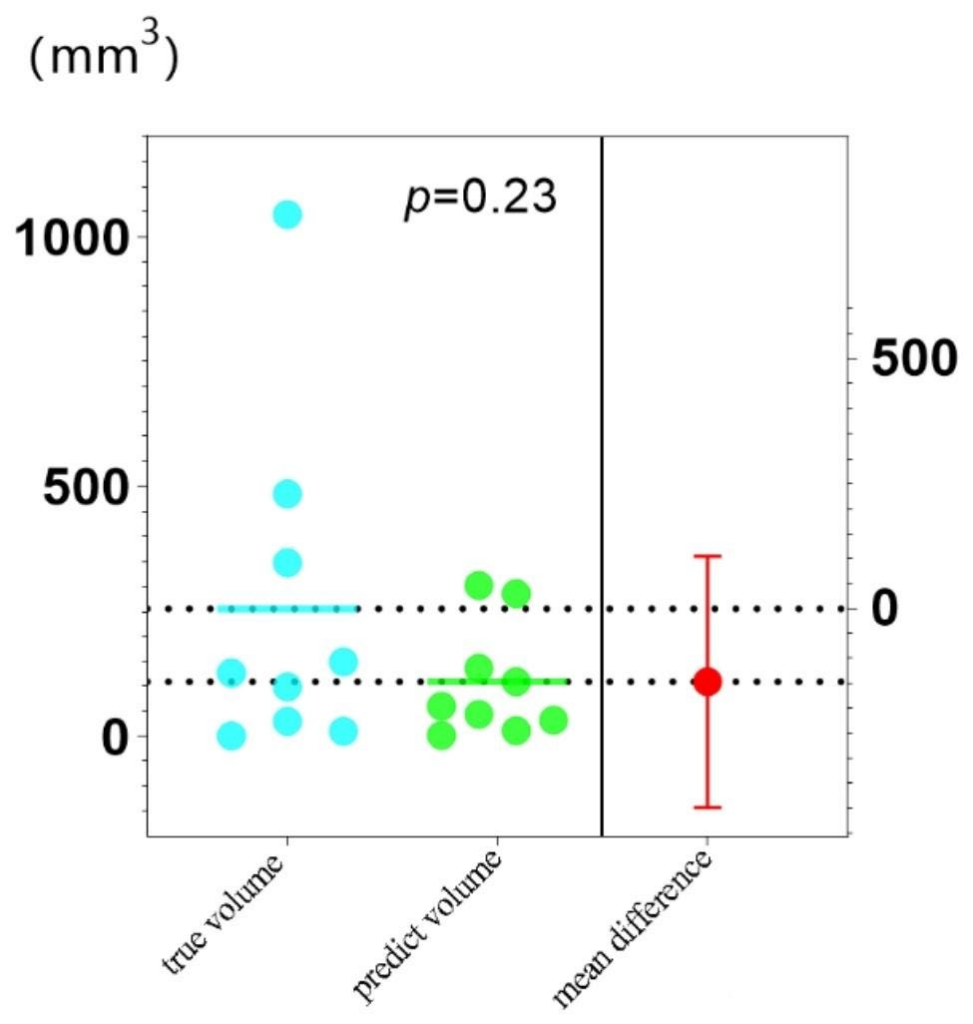
Comparison of manually and automatically delineated tumor volumes in the external test set. The actual and predicted volumes were compared. Since the *P* value was 0.23, the difference between the two indicators was not statistically significant

While evaluating the above segmentation results, the quality of the 2D slices was primarily considered. Clinicians are more concerned with the volume of segmented slices. Therefore, we compared the volume of tumors obtained by automated segmentation with the real volume obtained by manual segmentation. With a DSC of 0.391, most tumors were not accurately segmented in automated segmentation, resulting in a relatively small tumor volume. Figures 2 and 3 indicate the difference in the tumor volume between manual and automated segmentations from various perspectives. The difference in tumor volume can impact treatment decisions and follow-up evaluations. Smaller automated volumes may lead to underestimation, affecting accurate assessment. Clinical interpretation might be affected due to potential inconsistencies in tumor delineation. Strategies to harmonize automated and manual volumes are crucial for reliable clinical insights.

## Discussion

In this study, the DSC value of the liver and liver tumor segmentations was > 0.9 and > 0.6, respectively, with UNet + + applied to multisequence-enhanced MRI. Additionally, the automated segmentation of the liver and liver tumor, respectively, saved > 160 and > 20 s on an average per case. Moreover, the threshold segmentation method reduced false-positive results. This algorithm can likely improve diagnostics and efficacy of MR imaging.

Few studies are conducted on the segmentation of the liver and liver tumors using multisequence-enhanced MRI. The baseline conditions of the patients enrolled in this study were consistent with those of real-world patients with liver cancer (Table 1). Zhou [32] evaluated the data set segmented by UNet + + using six public medical images, and the DSC of CT-based liver segmentation was > 0.9. In our study, in the automated segmentation of the liver, the DSC of the validation and internal testing sets was 0.91 and 0.92, respectively. In the subsequent segmentation of liver tumors, the DSC values in the internal testing set based on patients and lesions were 0.526 and 0.612, respectively. For further validation, an external test set was used, in which the DSC values of liver tumor segmentation based on patients and lesions were 0.611 and 0.687, respectively. The automated segmentation saved 25.3–44.5 s. For the internal testing and external sets, the time ratio between automated and manual segmentations was 1:16 and 1:17, respectively. Bousabarah et al. [33] applied UNet network structure to detect liver tumors using MRI sequences of three phases; mean DSC between automated and corresponding manual segmentations of lesions was 0.64/0.68 (validation/test) and 0.91/0.91 for the liver segmentations. Although the conclusions of Bousabarah et al. [33] are generally consistent with our results, their study did not perform external verification. In our study, we compared the difference between the real and the predicted volumes of liver tumors in the external validation set, and statistical analysis indicated that the difference was not significant (*P* = 0.23). Chen et al. achieved the DSC values of 0.92 and 0.75 for CT-based liver and liver tumor segmentations, respectively. In our study, the DSC of liver tumor segmentation was < 0.7, possibly because of the complicated MR imaging protocol and average tumor diameter of 2.2 cm. A meta-analysis [34] reported that dynamic contrast-enhanced MRI was 89% specific for detecting liver tumors of small size but was only 64% sensitive. Importantly, although both statistics were higher than the CT results, missed diagnosis is still possible, which would result in the training model being less effective in detecting small lesions. Supplementary Table 4 indicated that in the internal testing set, unsegmented lesions were observed in case nos. 2, 3, 9, and 11, in which all lesions were < 1 cm in diameter.

A high false positivity rate has always been a barrier to improve the accuracy of intrahepatic tumor segmentation. Despite its ability to detect lesions that can be overlooked by clinicians, erroneous segmentation of blood vessels as tumors is common. Supplementary Tables 4 and 6 indicated that the AFPRs of the internal testing and external test sets were 1 and 1.6, respectively, which were higher than those reported by Bousabarah et al. (0.75). This may be explained by the fact that in our study, the liver was automatically segmented, and the automatic detection was performed only on the liver contour, leaving the blood vessels in the liver unsegmented. Further, the detection results were again input into the model to perform intrahepatic tumor segmentation. The lesions exhibited high signal in the arterial phase and medium signal in T2WI. During image post-processing, the threshold was adjusted to reduce the false positivity rate. However, the signal intensity of the tumor in the arterial phase was close to that of a certain blood vessel, which was difficult for the computer to distinguish. False image registration between T2WI and arterial phase led to mismatched signals. In total, 2 lesions were present in segment 4 (Fig. 2). Their signal was significantly different from that of the surrounding liver tissue, which could be misdiagnosed as a tumor by computer. However, the false-positive lesions were right next to the real tumor (Fig. 2). Considering that this study is based on 2D image modeling, the connection was not considered between layers in the slicing area where the liver tumors were located. Additionally, the oversegmentation of the liver may lead to the introduction of extrahepatic interference, resulting in the identification of some tissues outside the liver as lesions. Although the automated segmentation covered most of the liver tumor area, some extrahepatic tissue was misidentified as liver tumor (Fig. 2). Nevertheless, the false positivity rate in this study is acceptable in clinical practice.

This study has some limitations. It must be noted that sufficient amount of publicly available data was not used in this study. Comparisons with previous studies that used different data sets may cause performance errors. Dynamic contrast-enhanced MRI is a complex technique, and subtle changes in the imaging protocol may affect the performance. Prospective, multicenter studies are needed to further verify the generalization ability of the model. Accuracy of the registration determines the performance of a model; therefore, further research is necessary on more advanced registration methods. This study used only 2D images and did not effectively use spatial context information.

In this study, we employed the UNET + + architecture by integrating T2-weighted and T1-enhanced sequences to enhance the efficiency of liver and intrahepatic tumor segmentation. This integration shows promise in reducing the time required for manual annotations by clinical professionals and creating better conditions for advanced imaging genomics research. While the automatic tumor segmentation accuracy still requires improvement, the approach of combining these two sequences may offer novel insights for further enhancing segmentation precision.

### Electronic supplementary material

Below is the link to the electronic supplementary material.

Supplementary Material 1

Supplementary Material 2

## Data Availability

Data supporting the findings of this study can be obtained from the corresponding author upon request. Program source code address: https://github.com/hicccp/liver-and-tumor-auto-segmentation.
