# Supplementary material for: A deep-learning approach for segmentation of liver tumors in magnetic resonance imaging using UNet++
Source: BMC Cancer. 2023 Nov 3;23:1060. doi: 10.1186/s12885-023-11432-x (PMC10623778; doi:10.1186/s12885-023-11432-x)
Supplement: Supplementary file 1 — Supplementary Material 1 [file 12885_2023_11432_MOESM1_ESM.docx]

**Supplement Tables**

Suppl Table 1. Liver and liver tumor automatic segmentation hardware environment configuration.

| Project | parameter |
| --- | --- |
| CPU | AMD EPYC 7742 64-Core Processor |
| CPU numbers | 4 |
| CPU core numbers | 64 |
| Memory Capacity | 1T |
| system | Linux |
| GPU | A100-SXM4-40GB |
| GPU numbers | This project uses at most 2 GPUs: two GPUs for model training and only one GPU for model inference |

**Suppl Table** **2. Liver and liver tumor automatic segmentation software environment configuration.**

| python third-party packages | version | Role in this project |
| --- | --- | --- |
| torch | 1.9.0+cu111 | Deep Learning Framework |
| SimpleITK | 2.0.1 | Medical image file reading and writing and processing, etc. |
| scikit-image | 0.17.2 | Image preprocessing and postprocessing |
| scikit-learn | 0.23.2 | Statistics of indicators such as DICE |
| numpy | 1.19.5 | Numeral Calculations |

**Suppl Table** **3. Comparison of Liver Automatic Segmentation and Manual Segmentation in Internal Validation and Test Sets.**

| Patient number | validation set | | |  | Test set | | |
| --- | --- | --- | --- | --- | --- | --- | --- |
|  | Manual segmentation | auto-segmentation | DSC |  | Manual segmentation | auto-segmentation | DSC |
| 1 | 180.5 | 2.7 | 0.923 |  | 150.6 | 1.5 | 0.949 |
| 2 | 175.6 | 2.2 | 0.907 |  | 130.2 | 1.1 | 0.874 |
| 3 | 220.2 | 3.6 | 0.942 |  | 310.5 | 3.4 | 0.930 |
| 4 | 150.6 | 1.3 | 0.934 |  | 90.2 | 0.6 | 0.973 |
| 5 | 223.5 | 3.0 | 0.904 |  | 110.3 | 0.9 | 0.931 |
| 6 | 150.1 | 0.9 | 0.938 |  | 85.6 | 0.6 | 0.927 |
| 7 | 161.3 | 1.4 | 0.932 |  | 150.1 | 1.4 | 0.917 |
| 8 | 142.5 | 0.8 | 0.811 |  | 129.6 | 1.1 | 0.870 |
| 9 | 135.3 | 1.2 | 0.947 |  | 410.3 | 5.2 | 0.900 |
| 10 | 302.5 | 4.3 | 0.916 |  | 200.2 | 2.0 | 0.923 |
| 11 | 170.2 | 2.2 | 0.858 |  | 100.2 | 0.7 | 0.949 |
| mean value | 182.9 | 2.2 | 0.910 |  | 169.8 | 1.7 | 0.922 |

Note : The time unit is seconds.

**Suppl Table 4. Comparison of automatic segmentation and manual segmentation based on patient liver tumor in the test set.**

| Patient number | Manual segmentation | Auto-segmentations | DSC/case | Sensitivity | False positive |
| --- | --- | --- | --- | --- | --- |
| 1 | 30.5 | 2.0 | 0.449 | 1 | 2 |
| 2 | 31.3 | 1.7 | 0.595 | 0.5 | 0 |
| 3 | 100.3 | 8.0 | 0.71 | 0.667 | 1 |
| 4 | 30.5 | 1.8 | 0.305 | 1 | 2 |
| 5 | 30.3 | 1.2 | 0.617 | 1 | 2 |
| 6 | 40.2 | 0.9 | 0.253 | 1 | 2 |
| 7 | 31.4 | 2.2 | 0.82 | 1 | 1 |
| 8 | 56.3 | 1.9 | 0.443 | 1 | 2 |
| 9 | 90.3 | 7.6 | 0.45 | 0.4 | 2 |
| 10 | 30.3 | 3.0 | 0.355 | 1 | 1 |
| 11 | 50.2 | 1.2 | 0.79 | 0.5 | 0 |
| Mean value | 47.4 | 2.9 | 0.526 | 0.824 | 1 |

Note: This table is based on statistics for each case

**Suppl Table 5. DSC performance on the test set based on automatic segmentation of each tumor.**

| Patient number | Tumor number | DSC |
| --- | --- | --- |
| 1 | 1 | 0.473 |
| 2 | 1 | 0.595 |
| 2 | 2 | 0.000 |
| 3 | 1 | 0.741 |
| 3 | 2 | 0.836 |
| 3 | 3 | 0.000 |
| 4 | 1 | 0.327 |
| 5 | 1 | 0.735 |
| 6 | 1 | 0.547 |
| 7 | 1 | 0.833 |
| 8 | 1 | 0.484 |
| 8 | 2 | 0.437 |
| 9 | 1 | 0.681 |
| 9 | 2 | 0.000 |
| 9 | 3 | 0.656 |
| 10 | 1 | 0.452 |
| 10 | 2 | 0.590 |
| 11 | 1 | 0.791 |
| 11 | 2 | 0.000 |
| Mean Value ( based on real tumors ) |  | 0.526 |
| Mean value( only for automatically segmented tumors ) |  | 0.612 |

**Suppl Table 6. Comparative performance of automatic and manual segmentation of liver tumors on an external validation set.**

| Patient number | Manual segmentation | Auto-segmentation | DSC/case | sensitivity | false positive |
| --- | --- | --- | --- | --- | --- |
| 1 | 40.2 | 6.4 | 0.868 | 1.000 | 2.0 |
| 2 | 35.2 | 6.0 | 0.768 | 1.000 | 2.0 |
| 3 | 30.5 | 4.2 | 0.363 | 1.000 | 2.0 |
| 4 | 20.7 | 1.1 | 0.916 | 1.000 | 0.0 |
| 5 | 15.3 | 0.7 | 0.653 | 1.000 | 1.0 |
| 6 | 35.6 | 6.0 | 0.391 | 1.000 | 3.0 |
| 7 | 34.7 | 5.9 | 0.502 | 1.000 | 2.0 |
| 8 | 32.7 | 5.3 | 0.508 | 1.000 | 0.0 |
| 9 | 24.6 | 2.6 | 0.515 | 1.000 | 2.0 |
| Mean Value | 29.5 | 4.2 | 0.611 | 1.000 | 1.6 |

**Suppl Table** 7**. DSC results on an external test set for liver tumor segmentation.**

| Patient number | tumor number | DSC |
| --- | --- | --- |
| 1 | 1 | 0.879 |
| 2 | 1 | 0.855 |
| 3 | 1 | 0.513 |
| 4 | 1 | 0.916 |
| 5 | 1 | 0.870 |
| 6 | 1 | 0.391 |
| 7 | 1 | 0.433 |
| 8 | 1 | 0.508 |
| 9 | 1 | 0.820 |
| mean |  | 0.687 |
