# Supplementary material for: A deep-learning approach for segmentation of liver tumors in magnetic resonance imaging using UNet++
Source: BMC Cancer. 2023 Nov 3;23:1060. doi: 10.1186/s12885-023-11432-x (PMC10623778; doi:10.1186/s12885-023-11432-x)

**Supplement Figures**

**Suppl Fig 1.** Liver and Tumor Automatic Segmentation Pipeline. In the first stage, UNet++ network structures were used to input preprocessed MRI images and output liver masks. In the second stage, a tumor mask was produced from the liver image segmented in the first stage. By using the output from the first stage liver semantic segmentation image as input to the second stage network, it may be possible to eliminate the interference of pixels outside the liver that may interfere with tumor semantic segmentation in the liver, which will improve tumor semantic segmentation accuracy. Note: The blue dotted line in the picture is the boundary automatically drawn by the computer. Process for combining deep learning and post-processing (thresholding) to reduce false positive rates


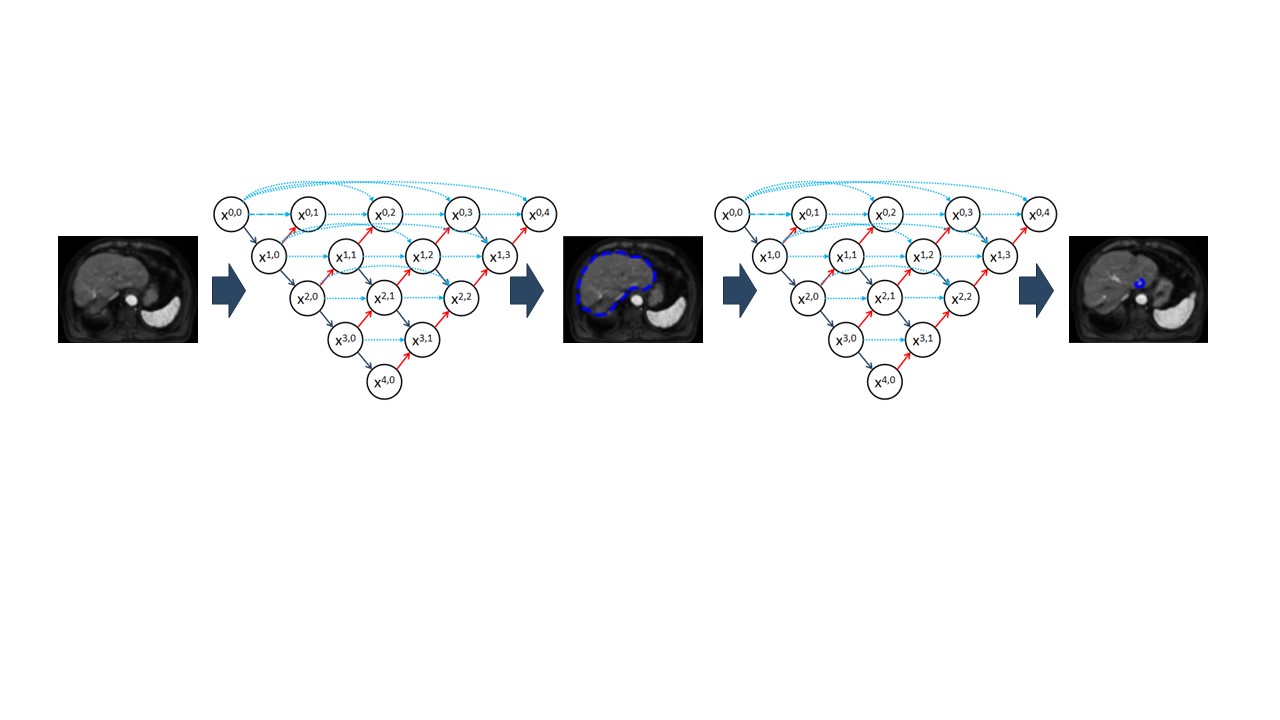


**Suppl Fig 2.** If the mean value was less than the threshold (the threshold was determined to be 0.5 according to training set statistics), it indicated that the independent connection domain was a false positive, which should be deleted; otherwise, the independent connection domain would be retained. Finally, through morphological closure operation, small holes in the tumor mask were eliminated, improving the tumor mask's prediction accuracy.

Best Threshold Selection Process.
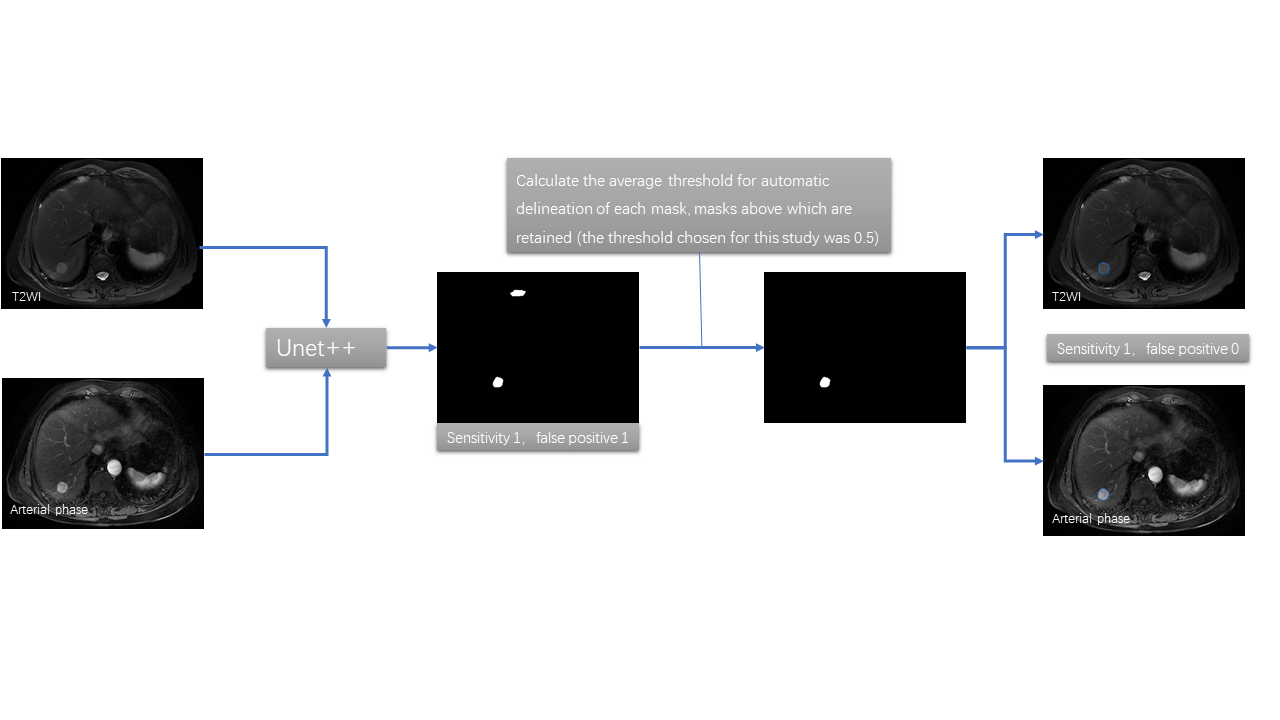


Suppl Fig 3. As part of the model training process, we determined the sensitivity, specificity,accuracy, and F1-score of the model output under various thresholds, and ultimately determined that 0.5 was the optimal threshold. The worst patient in the internal test set for liver segmentation (No. 8).


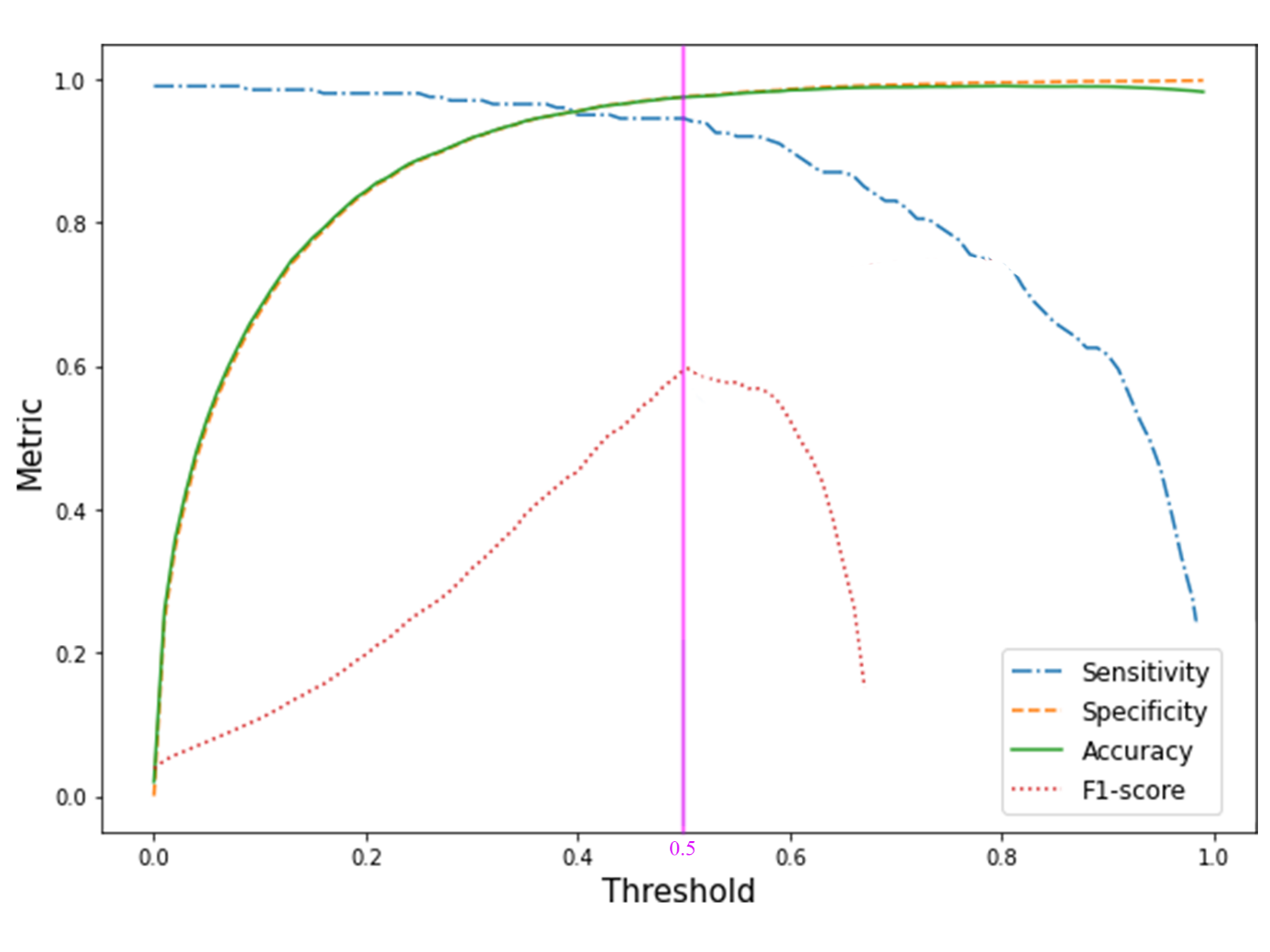


**Suppl Fig 4.** Blue solid lines represent the ground truth, and red dotted lines represent automatic segmentation. In this case, the patient has been diagnosed with liver cancer after undergoing surgery. It is important to note that the red solid line covers all of the blue solid lines, while the excess is mostly located outside the liver. In this case, the DSC value is 0.87. The best patient in the internal test set of liver segmentation (No. 4).


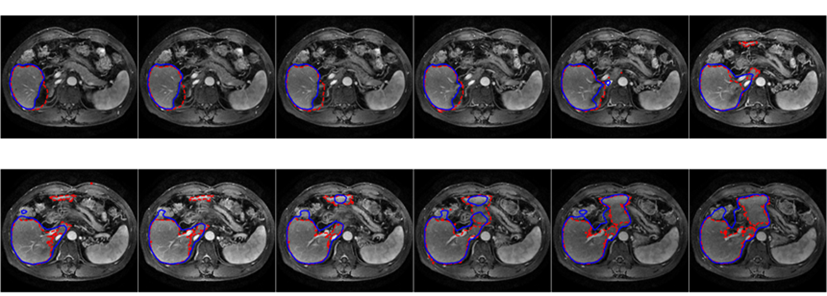


**Suppl Fig 5.** The blue solid line is ground truth, and the red dashed line is automatically drawn. The automatic delineation of patient No. 4 is almost identical to the manual delineation, and the DSC value is 0.973. The worst patient in the internal test set for tumor segmentation (No. 6).


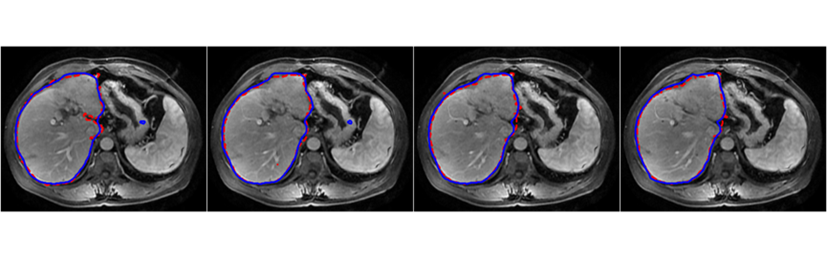


**Suppl Fig 6.** The blue solid line is the ground truth, and the red dotted line is the automatic segmentation. Figures 7-d to 7-i indicate that there are 2 false-positive lesions in segment 4 of the liver, and Figures 7-a to 7-c Of the four tumors, only one lesion was accurately segmented, with a DSC value of 0.253.

The best patient in the internal test set of liver segmentation (No. 7).


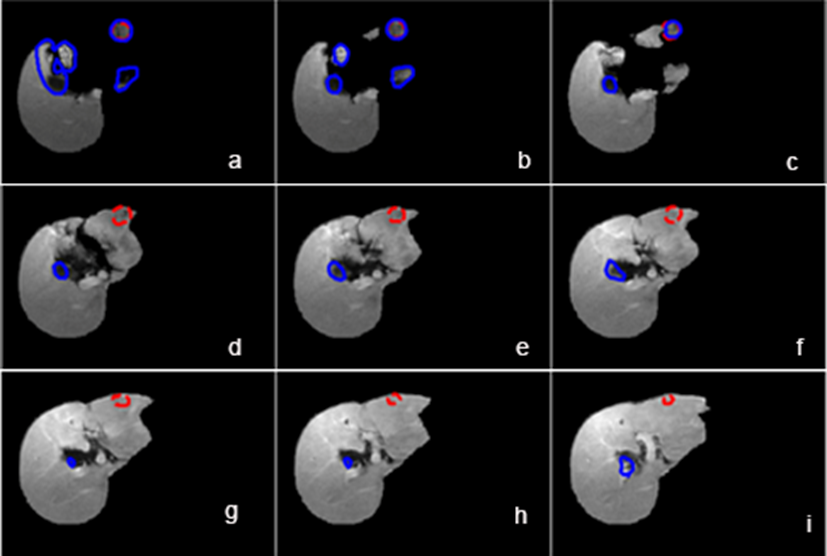


**Suppl Fig 7.** In this case, there is a false positive lesion at the edge of the lesion, and the automatic segmentation of the real lesion is in full agreement with the gold standard, DSC 0.82, false positive rate 1. External test set No. 1 tumor automatic segmentation results display.


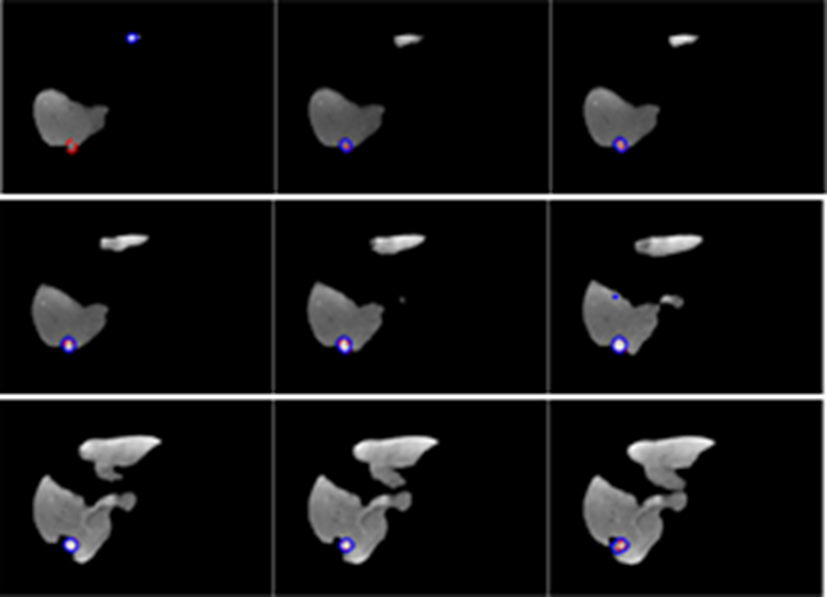


**Suppl Fig 8.** The red dashed line in the external test set No. 1 covers most of the blue solid line, DSC 0.879, but part of the extrahepatic tissue was mis-segmented as tumor.


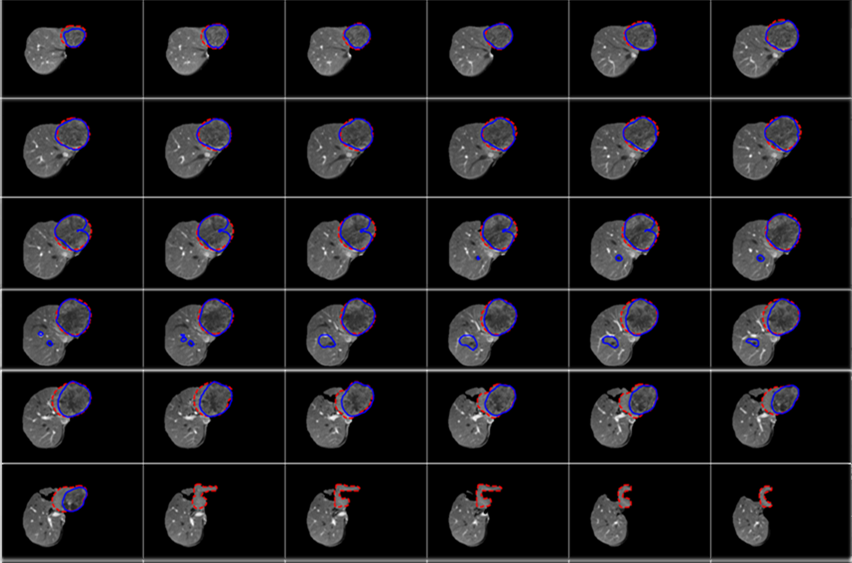

Supplement: Supplementary file 2 — Supplementary Material 2 [file 12885_2023_11432_MOESM2_ESM.docx]
